# Exploring and Characterizing Patient Multibehavior Engagement Trails and Patient Behavior Preference Patterns in Pathway-Based mHealth Hypertension Self-Management: Analysis of Use Data

**DOI:** 10.2196/33189

**Published:** 2022-02-03

**Authors:** Dan Wu, Xiaoyuan Huyan, Yutong She, Junbin Hu, Huilong Duan, Ning Deng

**Affiliations:** 1 College of Biomedical Engineering and Instrument Science Ministry of Education Key Laboratory of Biomedical Engineering Zhejiang University Hangzhou China; 2 The First Health Care Department The Second Medical Center & National Clinical Research Center for Geriatric Diseases Chinese People's Liberation Army General Hospital Beijing China; 3 Health Community Group of Yuhuan People's Hospital Kanmen Branch Taizhou China; 4 Alibaba–Zhejiang University Joint Research Center of Future Digital Healthcare Hangzhou China; 5 Binjiang Institute of Zhejiang University Hangzhou China

**Keywords:** hypertension, mobile health, patient behavior, engagement, data analysis

## Abstract

**Background:**

Hypertension is a long-term medical condition. Mobile health (mHealth) services can help out-of-hospital patients to self-manage. However, not all management is effective, possibly because the behavior mechanism and behavior preferences of patients with various characteristics in hypertension management were unclear.

**Objective:**

The purpose of this study was to (1) explore patient multibehavior engagement trails in the pathway-based hypertension self-management, (2) discover patient behavior preference patterns, and (3) identify the characteristics of patients with different behavior preferences.

**Methods:**

This study included 863 hypertensive patients who generated 295,855 use records in the mHealth app from December 28, 2016, to July 2, 2020. Markov chain was used to infer the patient multibehavior engagement trails, which contained the type, quantity, time spent, sequence, and transition probability value (TP value) of patient behavior. K-means algorithm was used to group patients by the normalized behavior preference features: the number of behavioral states that a patient performed in each trail. The pages in the app represented the behavior states. Chi-square tests, Z-test, analyses of variance, and Bonferroni multiple comparisons were conducted to characterize the patient behavior preference patterns.

**Results:**

Markov chain analysis revealed 3 types of behavior transition (1-way transition, cycle transition, and self-transition) and 4 trails of patient multibehavior engagement. In perform task trail (PT-T), patients preferred to start self-management from the states of task blood pressure (BP), task drug, and task weight (TP value 0.29, 0.18, and 0.20, respectively), and spent more time on the task food state (35.87 s). Some patients entered the states of task BP and task drug (TP value 0.20, 0.25) from the reminder item state. In the result-oriented trail (RO-T), patients spent more energy on the ranking state (19.66 s) compared to the health report state (13.25 s). In the knowledge learning trail (KL-T), there was a high probability of cycle transition (TP value 0.47, 0.31) between the states of knowledge list and knowledge content. In the support acquisition trail (SA-T), there was a high probability of self-transition in the questionnaire (TP value 0.29) state. Cluster analysis discovered 3 patient behavior preference patterns: PT-T cluster, PT-T and KL-T cluster, and PT-T and SA-T cluster. There were statistically significant associations between the behavior preference pattern and gender, education level, and BP.

**Conclusions:**

This study identified the dynamic, longitudinal, and multidimensional characteristics of patient behavior. Patients preferred to focus on BP, medications, and weight conditions and paid attention to BP and medications using reminders. The diet management and questionnaires were complicated and difficult to implement and record. Competitive methods such as ranking were more likely to attract patients to pay attention to their own self-management states. Female patients with lower education level and poorly controlled BP were more likely to be highly involved in hypertension health education.

## Introduction

### Background

Hypertension is a serious medical condition that affects health-related quality of life and increases the risks to the heart, brain, kidney, etc [1]. Controlling hypertension requires patients to achieve management goals by insistently adhering to long-term self-management plans, including regularly checking blood pressure (BP), taking medication, being physically active on a regular basis, eating more fruit and vegetables, and reducing alcohol consumption, etc. These plans should be established based on hypertension management guidelines and guidance from health care providers [2].

The development of mobile technology has promoted the implementation of out-of-hospital mobile health (mHealth) services [3-6]. Extensive evidence supports the effect of mHealth services in disease control [4,5,7-11], including promoting patient engagement in health care services and helping patients develop positive behavior in their daily self-management [12-16]. In comparison with transitional hypertension management methods, mHealth services can effectively improve patient engagement in hypertension self-management [17,18]. Cechetti et al [19] designed an mHealth app with a gamification method for hypertension management, which can effectively promote patient engagement in their self-management. However, while recent studies have demonstrated the effectiveness of some mHealth services, others have performed poorly [12-15]. There have been mixed results for using mHealth services to support patient self-management of hypertension in the community [20,21]. Thus, hypertension mHealth services pose new design challenges in management strategies, partly because the mechanism by which patients engage in their self-management is not clear.

Patient engagement is a broad concept that combines patient activation with interventions of health care services designed to increase activation and promote positive patient behavior [22]. Positive self-management behavior of patients engaging in mHealth services is essential for bringing an improvement in health outcomes [12,17,18,23-26]. Goyal et al [27] found a significant relationship between an increased number of daily blood glucose readings and improved glycated hemoglobin. Toto-Ramos et al [28] found that hypertensive patients with sustained engagement in mHealth services experienced significant reduction in BP. Thus, understanding self-management behavior of real-world patients in their daily lives can help to reveal patient behavior in natural settings. Compared to attracting patients from clinical trials who are more likely to overcome the burden associated with research work [29], this provides deeper insight into real patient self-management behaviors.

Hypertension management requires long-term efforts, and patient behavior in hypertension management is dynamic and continuous [30,31]. Understanding the longitudinal characteristics of patients engaged in self-management is important for long-term successful management. Moreover, there are many different dimensions of behavior with patients who engage in mHealth services [12,30,32,33], such as measuring, viewing, and recording, which are associated with individual inherent preferences and habits of patients [34-36]. Patient engagement behavior has been measured as amount, duration, breadth, and depth of using mHealth services [12]. Rahman et al [37] measured patient engagement by 3 key use features: duration and frequency of using the mHealth app plus the number of use records. Sanatkar et al [33] measured 5 use features of patient engagement: number of user log-ins, number of daily trackers used, numbers of learning activities started and completed, and number of reminders received. However, the multidimensional and dynamic behavioral processes that change over time cannot be captured simply by analyzing count data captured at 1 time point in the cross-sectional data analysis. Longitudinal change in patient multibehavior can be identified through analyzing the time series data. More comprehensive understanding of the multiple self-management behavior and individual behavior preferences of patients engaging in mHealth self-management over a long period requires further research. Data mining techniques have been successfully adopted in the analysis of longitudinal events, such as human behavior navigation [26,38-40], information search behavior [41], phase-type distribution in the health care industry [42], and lifetime health care costs [43]. These would allow us to infer the characteristics of patient behavior throughout the entire period of self-management.

### Objective

The aim of this study was to explore the trails of patient multibehavior engagement in the pathway-based mHealth hypertension self-management and identify the characteristics of patients with different behavior preferences. This included 3 objectives. The first objective was to discover the trails of patient multibehavior engagement. The second objective was to explore patient behavior preference patterns. The third objective was to identify the association between behavior preference patterns and the demographic and physiological characteristics. Identifying the multidimension and longitudinal characteristics of patient behavior within the mHealth hypertension management offers new opportunities for personalizing management goals and plans to reduce nonadherence and enhance possible effectiveness.

## Methods

### Description of the mHealth Hypertension Management App

**Figure 1:**
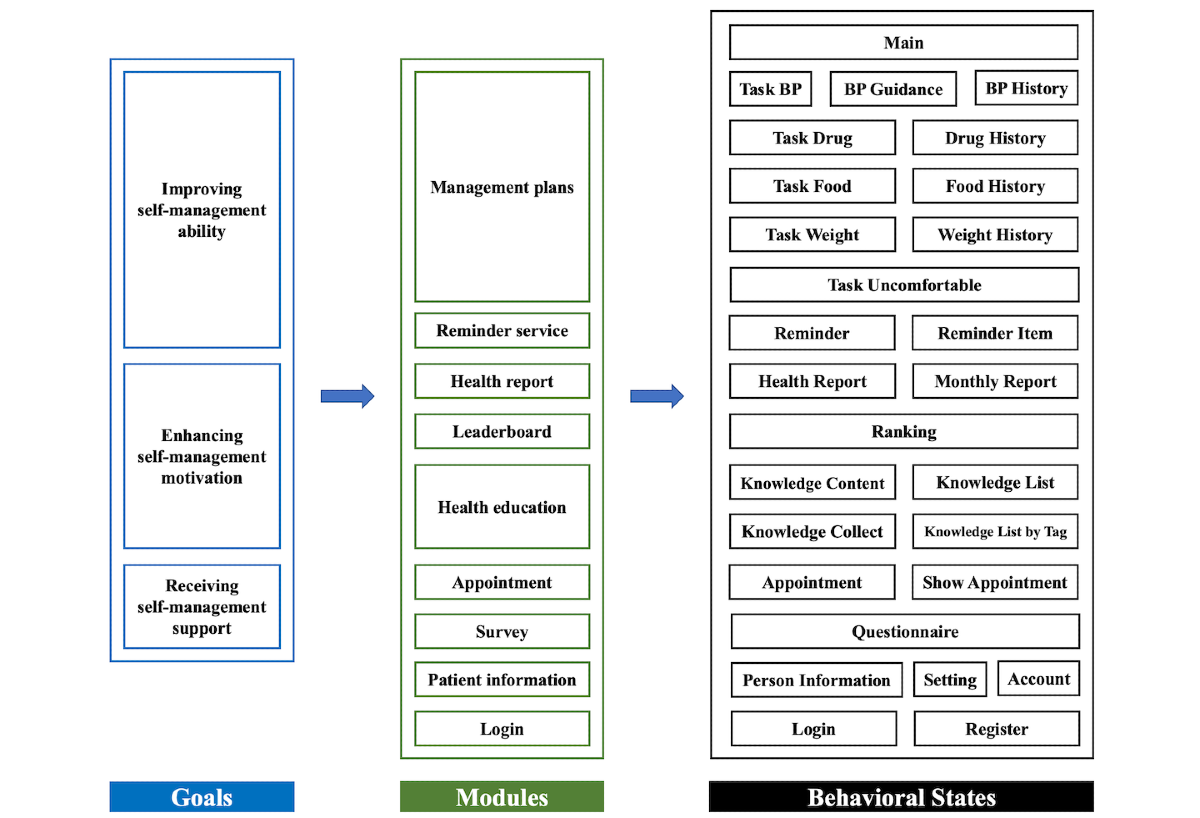
Components of pathway-based mHealth hypertension self-management. BP: blood pressure.

We used data from the Blood Pressure Assistant, a pathway-based hypertension self-management app in the Digital Care Study for Hypertension Management [44]. The app was designed in accordance with a customized care pathway in compliance with the Chinese guideline for hypertension management [45] and was available for patients in the General Hospital of Ningxia Medical University. The care pathway involves 2 roles in hypertension management: health care providers and patients. The care pathway defines 3 goals for patients—improve self-management ability, enhance self-management motivation, and receive self-management support (see Figure 1)—and comprises 9 modules and 28 behavioral states (see Multimedia Appendix 1 for the detailed behavioral states). The 9 modules generate intervention plans and patient self-management plans. The term of state comes from the Markov decision process, a mathematical model of sequential decision. The behavioral state is a description of the patient’s behavior in a hypertension self-management environment or scene and is expressed by pages in the mHealth app. In this paper, we chose to use state to represent the patient behavior.

In this care pathway, patient actions on different pages of the app reflect specific patient behaviors. The behavioral states are represented by different pages in the app. Through the app, each patient registers and enters basic demographic information, after which the patient is assigned to a health care provider who enrolls patients they manage into the mHealth hypertension management program at an online community [45]. The health care provider is responsible for formulating a tailored management plan, reviewing patients’ uploaded data, and conducting follow-up. Patients can use the app to seek help from health care providers, check their management plans, and record self-management data including BP monitoring, medication, physical activity, and diet. These patient behaviors are represented on the appointment, main, task BP, task drug, and other pages of this mHealth app. Health care providers can track current BP readings of patients through a web-based platform to adjust management plans and use mobile phones for patient follow-ups to assist in BP control.

### Data Collection

#### Ethics

Ethical approval was granted by the ethics committee for conducting human research at the General Hospital of Ningxia Medical University (NXMU-GH-2017-273). All patients in this study signed the informed consent forms for their anonymized data to be used in routine evaluations to monitor and improve health care services.

#### Sample

Since this app was launched in December 2015, 1159 patients have used it to self-manage their BP. We selected patients based on the following inclusion criteria: aged 18 to 80 years, diagnosed with hypertension, performed self-management between December 28, 2016, and July 2, 2020 (the main functions of the app were consistent during this period, ensuring patient behavior was not affected by the changes in app functions).

#### Data Extraction

All data were stored and extracted from the server of the Blood Pressure Assistant, which contains the self-management plans, demographic information, uploaded self-management data from patients, and follow-up records of health care providers. We extracted 3 types of data from the database: demographics, physiological records, and patient use records. Demographics included patient identification, data of birth, gender (male or female), and education level (below high school, high school, university and above). Physiological records included patient identification, systolic blood pressure (SBP), diastolic blood pressure (DBP), heart rate (HR), and uploaded date (year, month, day, minute, and second). Patient use records included patient identification, page name, time page is entered and exited, and stay time. These data help determine longitudinal and multidimensional behavioral characteristics of hypertensive patients and characteristics of patients with different behavior preferences.

### Data Analysis

#### Identifying Patient Multibehavior Engagement Trails

**Figure 2:**
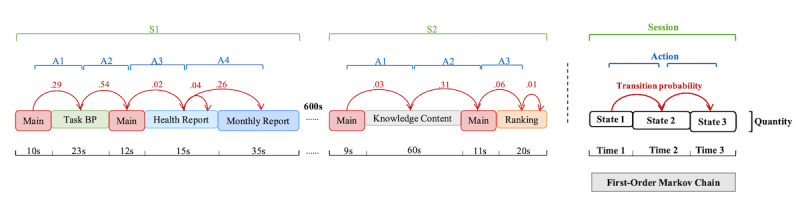
Example of patient multibehavior engagement trail represented by first-order Markov chain. BP: blood pressure.

Patient multibehavior engagement trails were indicated by the type, quantity, time spent, sequence, and transition probability value (TP value) of patient behavioral states (see Figure 2). The time spent and sequence were calculated from the time pages were entered and exited.

The data of patient use behavior were analyzed through first-order Markov chain analysis, a method to model stochastic processes, which is suitable for analyzing user interaction with mHealth apps [46,47]. In the mHealth field, interaction between users and apps is determined through a process of connecting multiple continuous actions together. In the case of this study, we assumed that a next state depends only on the current state and not on the history of previous states. Thus, we used the first-order Markov chain called a memoryless model, containing the state space S , action, session, and transition matrix P. The discrete state space S was defined by the *n* different behavioral states (represented by different pages in the app): S={*s*_1_,...,*s_n_*}. A start state *s*_0_ and an exit state *s_n_*_+1_ were created when the patient started and left the app. The action was defined by the transition from one behavioral state to another (*s_i_*→*s_j_*). The session was discriminated by the time interval between 2 consecutive states greater than 600 seconds (*s_i_*–*s_j_*>600 *s*). The transition matrix P contained each element on row *i* and column *j* (*p_i,j_*) and indicated the transition probability that a patient moves from state *s_i_* to state *s_j_*. The transition probability *p_i,j_* was defined as



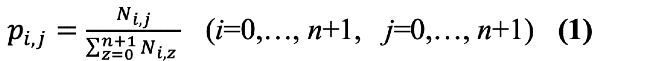



where *N_i,j_* was the number of transitions *s_i_*→*s_j_*. The quantity and time spent of the behavioral state are represented by the width and length of the rectangle (see Figure 2).

The transition matrix P was displayed by a heat map, which allowed us to understand the characteristics of patient multibehavior transition. Analysis was carried out using Python (version 3.8, Python Software Foundation). The 1% relative quantity of states and the 0.15 transition probability were used as cutoff values to improve readability. We then combined quantity, time, transition probability, and the pathway to plot the main trails of patient multibehavior engagement.

#### Cluster Analysis of Patients With Different Behavior Preferences

Cluster analysis was conducted to group patients according to patient behavior preference features: the number of behavioral states that a patient performed in each trail. First, we normalized the behavior preference features of each patient. The sum of behavior preference features of each patient for different behavior trails was 1. A K-means algorithm was then used to cluster patients through these normalized patient behavior preference features. Euclidean distance was used to calculate the similarity of features between patients. Finally, we used a silhouette score to determine the optimal number of clusters. Higher silhouette scores indicate tighter clusters, where each cluster is completely separate from the others.

#### Characterizing the Clusters

An optimal clustering result was reached based on the silhouette score of different numbers of clusters. For each cluster, we analyzed the demographic features (age, gender, and education level), and physiological features (mean BP [SBP and DBP] and mean HR). Statistical analysis was conducted in SPSS (version 24, IBM Corp). A chi-square test was performed to evaluate the statistical significance of associations between the clusters and discrete variables (gender and education level). A z-test was used to conduct pairwise comparisons of the differences among the proportion of discrete variables between the clusters. Analyses of variance (ANOVAs) were used to evaluate the statistical significance of associations between the clusters and continuous variable (age, BP, and HR). Bonferroni multiple comparisons were further conducted to examine the differences between the clusters. The cutting value (*P*≤.05) was used to determine whether the difference was statistically significant.

## Results

### Patient Multibehavior Engagement Trails

#### Quantity and Time Characteristics of Each Behavioral State

We restricted analysis to the remaining 863 patients, with 295,855 records from the app. Tables 1 and 2 depict the quantity and time spent in each behavioral state. It was clear that the main state (125,487 [42.42%]; 1,745,286 s [13.91 s]) was visited most in quantity and total time spent; it was the default starting page for a session. The relative number of 14 states exceeded 1%, and the mean time spent of 12 states exceeded 20 seconds. In 5 self-management tasks, the state of task BP (48,935 [16.54%]; 1,253,924 s [25.62 s]) was visited more, and the total time spent was also longer. The mean time spent on task food (149,164 s [35.87 s]) state was more than other self-management tasks. The states of ranking (7856 [2.66%]) and knowledge content (7330 [2.48%]) had relatively high quantities of visits. The states of knowledge content (615,430 s [83.96 s]) and show appointment (261,371 s [162.75 s]) had relatively long mean time spent. In 9 modules, the mean time spent in management plans (contained 5 self-management task parts: 230.21 s) was longer than health education (contained 4 knowledge parts: 124.06 s) and appointment (contained 2 appointment parts: 168.72 s).

**Table 1:** Quantity of each behavioral state.

Behavioral state^a^	Quantity, n (%)
Main	125,487 (42.42)
Task blood pressure	48,935 (16.54)
Task weight	33,560 (11.34)
Task drug	26,509 (8.96)
Ranking	7856 (2.66)
Knowledge content	7330 (2.48)
Blood pressure history	6070 (2.05)
Knowledge list	4871 (1.65)
Task food	4159 (1.41)
Questionnaire	4128 (1.40)
Health report	4055 (1.37)
Reminder item	3287 (1.11)
Appointment	3209 (1.08)
Weight history	3150 (1.06)
Reminder	2434 (0.82)
Drug history	2286 (0.77)
Show appointment	1606 (0.54)
Task uncomfortable	1345 (0.45)
Person information	1267 (0.43)
Monthly report	1144 (0.39)
Knowledge collect	1065 (0.36)
Knowledge list by tag	742 (0.25)
Blood pressure guidance	721 (0.24)
Setting	207 (0.07)
Food history	174 (0.06)
Account	125 (0.04)
Log-in	113 (0.04)
Register	20 (0.01)

^a^Descending by quantity (relative) of behavioral states.

**Table 2:** Time spent in each behavioral state.

Behavioral state^a^	Time spent (total), s	Time spent (mean), s
Show appointment	261,371	162.75
Knowledge content	615,430	83.96
Task food	149,164	35.87
Monthly report	39,590	34.61
Register	639	31.95
Blood pressure history	185,240	30.52
Blood pressure guidance	19,772	27.42
Task blood pressure	1,253,924	25.62
Log-in	2575	22.78
Task drug	600,104	22.64
Task weight	746,268	22.24
Knowledge list	100,995	20.73
Ranking	154,468	19.66
Food history	3391	19.49
Task uncomfortable	23,474	17.45
Account	2110	16.88
Weight history	52,218	16.58
Reminder item	52,493	15.97
Main	1,745,286	13.91
Knowledge list by tag	10,129	13.65
Health report	53,730	13.25
Drug history	28,309	12.38
Questionnaire	31,579	7.65
Setting	1451	7.00
Person information	7779	6.14
Appointment	19,157	5.97
Knowledge collect	6094	5.72
Reminder	8426	3.46

^a^Descending by time spent (mean) of behavioral states.

#### Patient’s Behavior Transition Matrix

**Figure 3:**
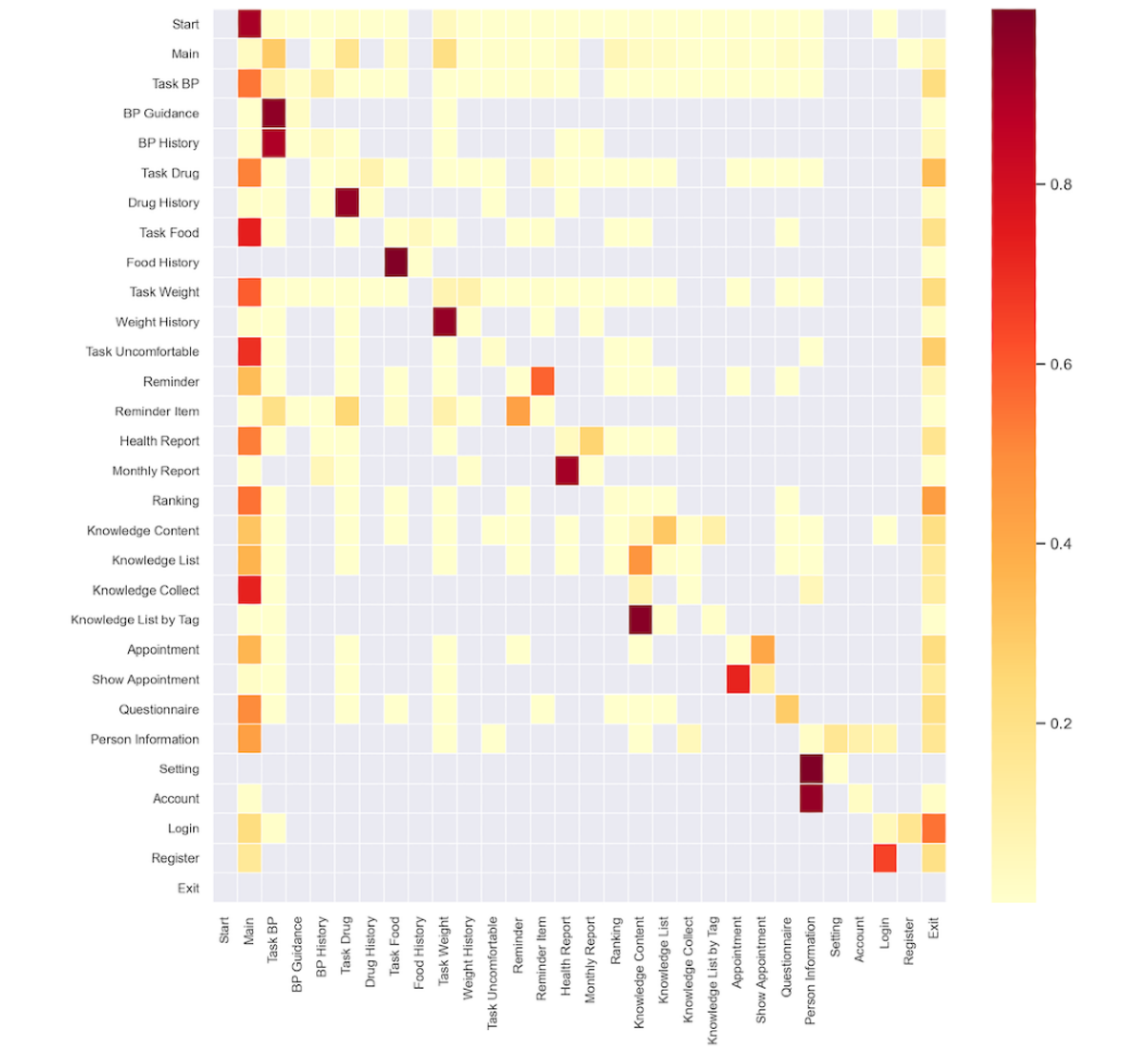
Transition matrix of patient multibehavior engagement. Probability of transitioning from behavioral state A[row] to B[column]. BP: blood pressure.

The heatmap was used to demonstrate the transition matrix of the Markov chain (see Figure 3). The various shades of color represent the probability of transition from one behavioral state to another, and the transparent color indicate that these 2 behavioral states cannot be transitioned. Horizontal and vertical coordinates indicate behavioral states. The rows summed up to 1. In most cases, the session started from the main state (0.91), and the transition probability from other behavioral states to the main state was also high, which was intended by design. When patients were in the main state, the states of task BP (0.29), task drug (0.18), and task weight (0.20) were the most visited among all behavioral states. Patients had a high probability if exiting the app from the following states: task drug (0.34), ranking (0.44), and log-in (0.55).

We found that the 1-way transition (*s_i_*→*s_j_*) from one behavioral state to another had a high probability in the following states: from BP guidance (0.96) or BP history (0.90) to task BP, from drug history to task drug (0.95), from food history to task food (0.99), from weight history to task weight (0.95), from reminder item to task BP (0.20) or task drug (0.25), from knowledge list by tag to knowledge content (0.98), and from account to person information (0.95). The cycle-transitions (*s_i_*⇔*s_j_*) between the 2 behavioral states had a high probability in the following states: between reminder and reminder item (0.58, 0.43), between health report and monthly report (0.26, 0.92), between knowledge content and knowledge list (0.31, 0.47), between appointment and show appointment (0.41, 0.73), between person information and setting (0.16, 1.00), and between log-in and register (0.17, 0.65). We found that the self-transition (*s_i_*
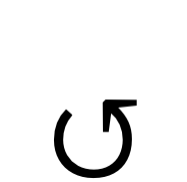
*s_i_*) in a behavioral state was very common, and a high self-transition can be seen in the questionnaire state (0.29).

#### Four Types of Patient Multibehavior Engagement Trails

**Figure 4:**
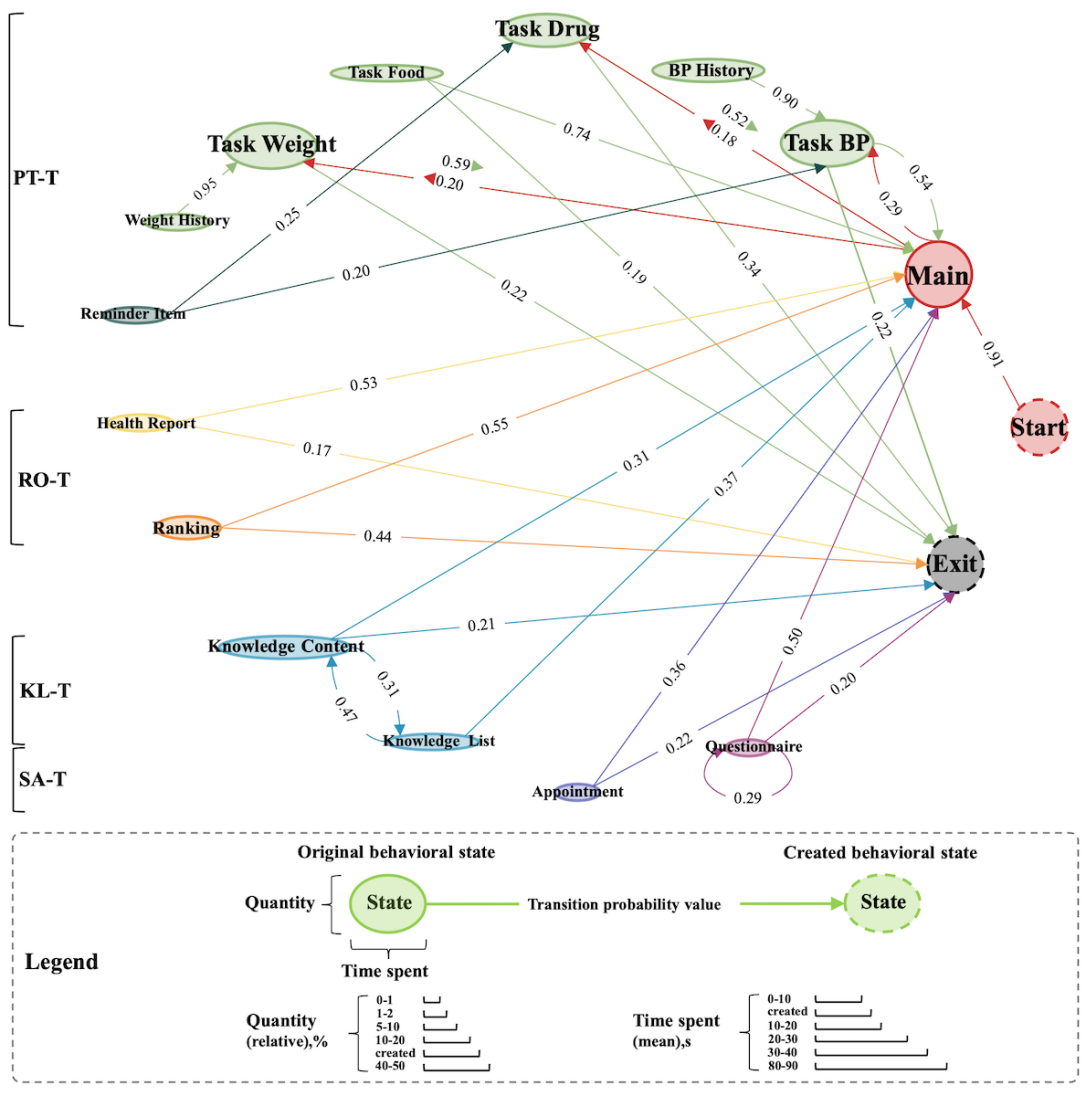
Four types of patient multibehavior engagement trails. BP: blood pressure.

There were 28 different types of behavior states and 283 possible behavior transitions in the original design in mHealth hypertension management app. The trails of patient multibehavior engagement in the pathway-based hypertension self-management were visualized by the main transitions between the different behavioral states (see Figure 4). The main trails of patient behavior included the type, quantity, time spent, sequence, and TP value. The size of a node indicated the quantity and time spent of behavioral states; a node or line of the same or similar color indicated a trail. Visual inspection of the transition between different behavioral states in mHealth hypertension self-management revealed several types of trails with different behavioral characteristics.

A first trail type can be labeled as a perform task trail (PT-T; green and dark green lines). After launching the app, patients go from the main state to the self-management task states to conduct management plans. Among the 5 self-management tasks, patients were more likely to transfer to the states of task BP, task drug, and task weight (TP value 0.29, 0.18, 0.20) than the states of task food, and task uncomfortable (TP value 0.03, 0.01). Moreover, some patients entered the states of task BP and task drug (TP value 0.20, 0.25) from the reminder item state, which happened when patients needed the app to remind themselves to measure BP and take medication on time. Patients also checked their BP history and weight history when they conduct the self-management tasks. After finishing the self-management tasks, there was a high probability that patients would return to the main state. The mean time spent was longer in the task food (35.87 s) state than that in the states of task BP, task drug, and task weight, and patients spent more time checking BP history (30.52 s) than weight history (16.58 s).

Second, a result-oriented trail (RO-T) can be distinguished (yellow and orange lines). Patients had a high probability in the states of health report and ranking in this trail, and the purpose was to check their self-management outcomes and their ranking among all hypertensive patients. The mean time spent in the ranking (19.66 s) state was longer than the health report (13.25 s) state, which showed that when competing with other patients, patients were more willing to spend time to understand the results of self-management. Eventually, this trail usually returned to the main state.

The third trail type was labeled a knowledge learning trail (KL-T; blue lines). In this trail, patients preferred to read the health education content. In addition, there was a high probability of cycle transition (TP value 0.47, 0.31) between the knowledge list state and the knowledge content state. This situation occurred when patients switched to a new piece of knowledge content after reading a previous piece of knowledge content. Patients spent an average of 83.96 s to reading a piece of knowledge content, and they spent an average of 20.73 s in the knowledge list state to select a piece of knowledge content to read. The end of this trail was sometimes the main state.

The last trail type was labeled a support acquisition trail (SA-T; purple and rese red lines). The states of questionnaire and appointment indicated the behavior of patients providing information to health care providers and seeking support from health care providers. There was a high probability of self-transition in the questionnaire (TP value 0.29) state, indicating the patients entered the questionnaire state, then switched to another app but did not exit the app, and then reopened the app on the questionnaire page. The end of this trail was sometimes the main state.

### Patient Behavior Preference Patterns

**Figure 5:**
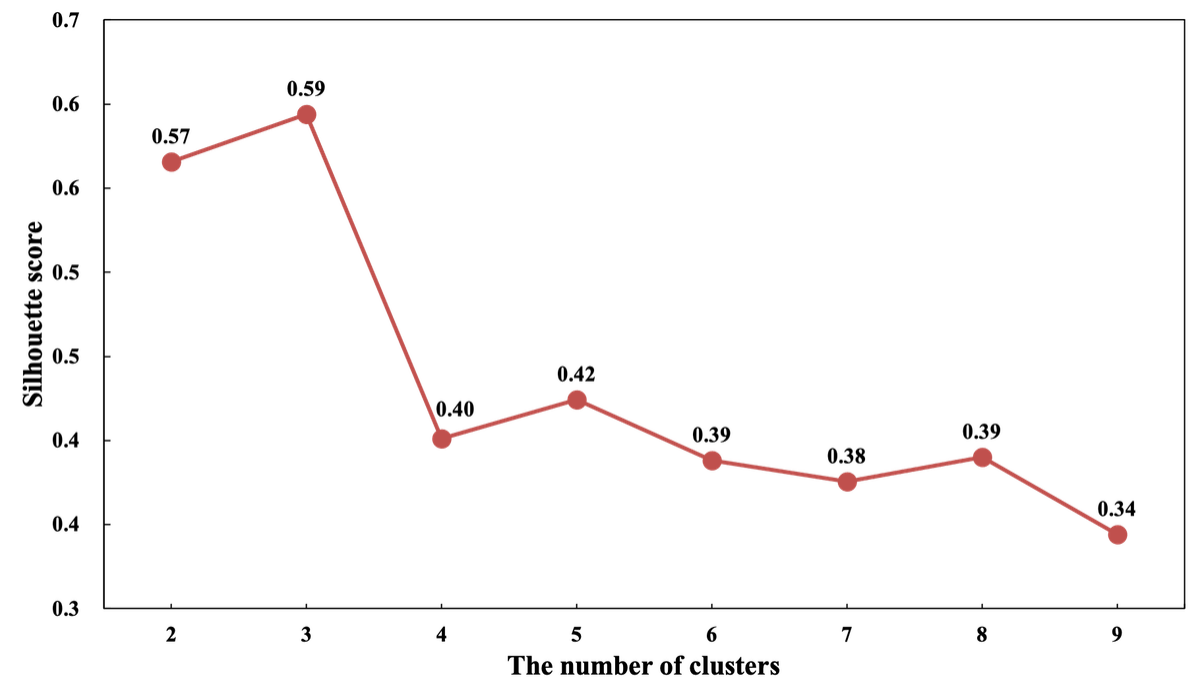
Comparison of the silhouette score for different number of clusters (range 2-9).

A total of 863 patients were selected for cluster analysis. We found that the silhouette score was the highest with 3 clusters of patients (see Figure 5). Hence, we accepted the 3-cluster output of K-means for further analysis. The patient behavior preferences in 4 trails were significantly different (PT-T *P*<.001, RO-T *P*=.06, KL-T *P*<.001, SA-T *P*<.001).

**Figure 6:**
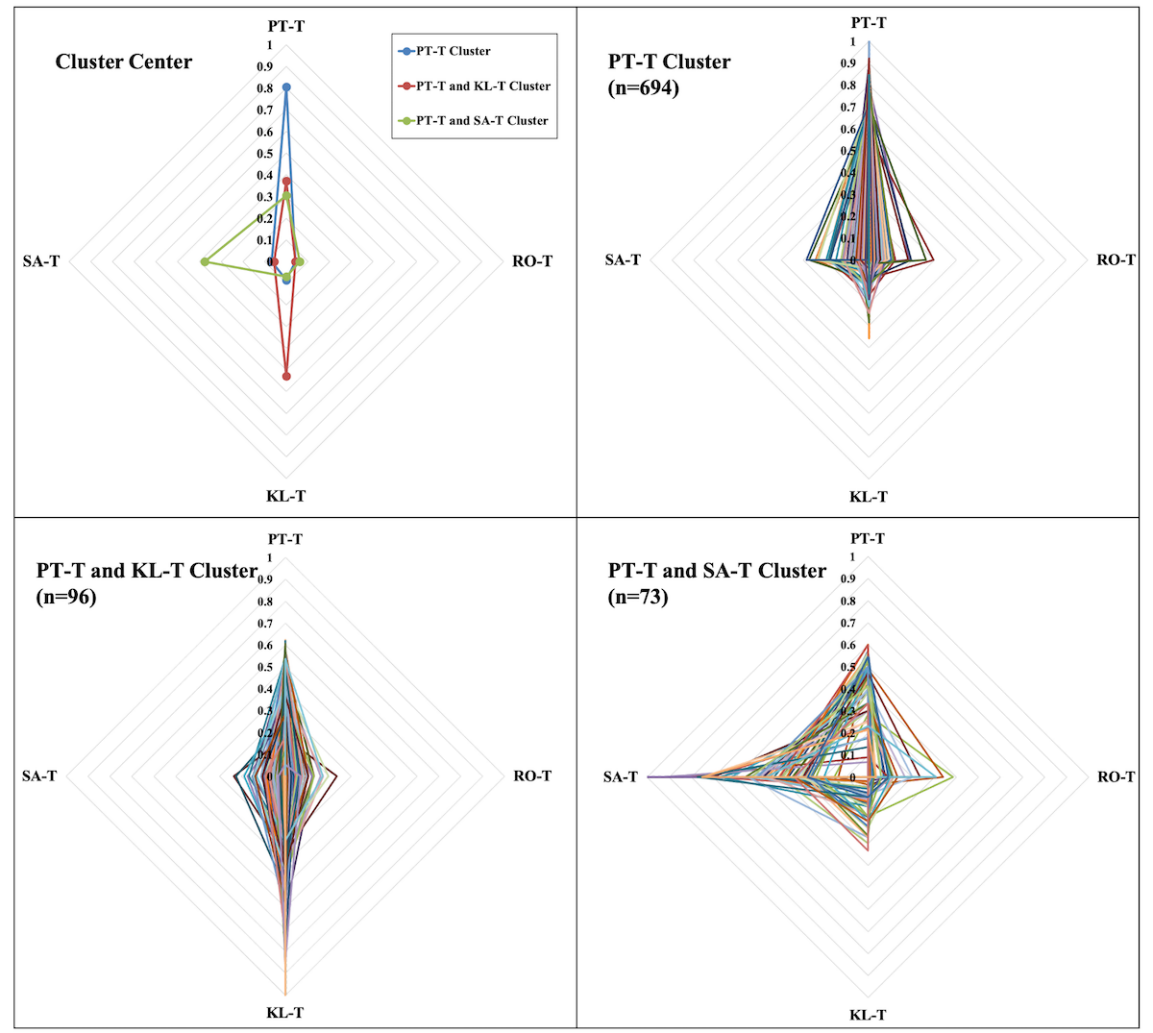
Three patterns of patient behavior preference.

There were 3 distinctive patterns of patient behavior preferences (see Figure 6). The sum of each patient’s behavior preferences for the 4 behavior trails was 1. The first cluster (PT-T) comprised 694 patients. Their behavior preference was particularly focused on PT-T (0.81), which indicated that this patient group preferred to perform self-management tasks. The second cluster (PT-T and KL-T) comprised 96 patients, who were active in PT-T (0.37) and KL-T (0.53). They were more likely to read knowledge about hypertension than to conduct self-management tasks. The third cluster (PT-T and SA-T) comprised 73 patients whose behavior preferences were PT-T (0.30) and SA-T (0.37). These patients were more willing to seek help from health care providers than to perform self-management tasks.

### Demographics and Physiological Characteristics of the 3 Behavior Preference Patterns

There were statistically significant associations between the behavior preference pattern and gender, education level, and BP, but there were not associations between the behavior preference pattern and age and HR (see Table 3). In the PT-T cluster and the PT-T and SA-T cluster, there were much more male patients than female patients, but the proportion of male and female patients was equal in the PT-T and KL-T cluster. Compared with the PT-T cluster, the PT-T and KL-T cluster was characterized by significantly fewer male patients, lower education level, and higher BP. The PT-T and SA-T cluster had significantly lower BP than the PT-T and KL-T cluster.

**Table 3:** Descriptive characteristics of the behavior preference pattern. Multiple comparisons of the 4 clusters (at the .05 level).

Characteristic		PT-T^a^ cluster (n=694)	PT-T and KL-T^b^ cluster (n=96)	PT-T and SA-T^c^ cluster (n=73)	*P* value
Age, mean (SD)		53.20 (9.93)	52.23 (9.99)	50.86 (10.34)	.13
**Gender, n (%)**		—^d^	—	—	.003
	Male	469 (67.58)	48 (50.00)^e^	49 (67.12)	—
	Female	225 (32.42)	48 (50.00)^e^	24 (32.88)	—
**Education, n (%)**		—	—	—	.05
	< High school	249 (35.88)	43 (44.79)	34 (46.57)	—
	High school	105 (15.13)	21 (21.88)	11 (15.07)	—
	≥ University	321 (46.25)	30 (31.25)^e^	25 (34.25)	—
	Don’t know	19 (2.74)	2 (2.08)	3 (4.11)	—
SBP^f^, mean (SD)		132.71 (15.43)	137.44 (17.96)^e^	130.77 (13.18)^g^	.02
DBP^h^, mean (SD)		86.46 (11.37)	90.60 (12.88)^e^	84.87 (11.29)^g^	.004
HR^i^, mean (SD)		72.41 (14.23)	73.20 (17.13)	73.23 (15.83)	.84

^a^PT-T: perform task trail.

^b^KL-T: knowledge learning trail.

^c^SA-T: support acquisition trail.

^d^Not applicable.

^e^Given cluster is significantly different from the PT-T cluster.

^f^SBP: systolic blood pressure.

^g^Given cluster is significantly different from the PT-T and KL-T cluster.

^h^DBP: diastolic blood pressure.

^i^HR: heart rate.

## Discussion

### Principal Findings

In this study, we used a stochastic model to describe longitudinal trails of patient multibehavior engagement with mHealth hypertension self-management and further analyzed the characteristics of patient groups with different behavior preferences. In a sample of 863 patients in mHealth hypertension self-management, we identified 3 types of behavior transition (1-way transition, cycle transition, and self-transition), 4 trails of patient multibehavior engagement (PT-T, RO-T, KL-T, and SA-T), and 3 behavior preference patterns (PT-T cluster, PT-T and KL-T cluster, and PT-T and SA-T cluster).

These insights revealed what actual patients do in daily hypertension self-management and how patients use the mHealth app to conduct self-management [32,43,47]. This may facilitate tailored and precise behavioral intervention strategies with specific content, methods, and time points for patient groups with specific behavior preferences. For example, for patient groups who measure and record BP but not take medication regularly, we will send a pop-up medication reminder in the app after completing the BP recording, rather than randomly and frequently reminding patients to measure BP and take medication. After they follow this reminder, we will provide rewards and push the knowledge about the advantages of regular medication to improve their adherence. This may promote more accurate chronic disease management strategies to achieve disease control.

### Patient Multibehavior Engagement Trails

The dynamic behavioral characteristics were identified by the transition probability between 2 continuous behaviors [39,47], which suggested the transitions in daily self-management behavior of hypertension patients. When patients started self-management, they were more likely to focus on BP, medications, and weight conditions. Patients prefer to pay attention to BP and medications from reminders, which can help them manage their conditions. Tao et al [48] found that the use of electronic reminders was associated with a significant improvement in patient adherence to medication. The cycle transition was often performed between 2 different behaviors of same module, such as reminder, health report, knowledge, and appointment, etc. This kind of behavior transition information gives us opportunities to provide specific behavioral interventions at an accurate time between 2 consecutive behaviors. In addition, self-transition was frequent when patients completed questionnaires. Patients were willing to engage in the questionnaire, but the time spent was very short. It may be because the questionnaire was too long and patients had no patience when completing it or the content of the questionnaire was difficult to understand. The clarity and conciseness of questionnaires should be improved so patients can better engage in this management part [49].

The trails of patient multibehavior engagement revealed the high probability sequences of patient behavior in mHealth hypertension self-management. This was a longitudinal and multidimensional process analysis rather than a cross-sectional analysis at a point in time. The 4 trails contained the main scenes of hypertension management in daily life [50] and described the behavioral conditions of patient self-management. In PT-T, for 5 types of self-management tasks of improving ability, patients preferred to start self-management by focusing on BP, medications and weight and spent more time on food. This may be attributed to diet management being more complicated and difficult to implement and record [51], which proposes a new challenge of how to improve the convenience of hypertension diet management. Samiul et al [52] proposed a network for automatic dietary monitoring that can gather food intake information through image, audio, and accelerometer sensors. By analyzing these data, the system can measure the type, volume, and nutrition of food, as well as the eating behavior of a person. Compared with weight history, patients spent more time reading BP history. In KL-T, patients usually chose different health knowledge contents from knowledge list to learn how to improve self-management ability. This knowledge may help patients broadly learn health concepts and behaviors [53]. In RO-T, patients read health reports to understand all aspects of their self-management, and knew the ranking of their self-management outcomes in an online hypertension community from the leaderboard. An interesting finding was that patients spent more energy on the rankings compared to reading health reports. This may suggest that gamification and competitive methods can effectively increase patient motivation and attention to their own self-management states [19,21,54]. In SA-T, patients sought help from health care providers through appointments, and completed the questionnaire to provide their own information and support research on hypertension management. These findings helped us dive into the daily self-management of actual patients and deeply understand patient behavioral characteristics through a longitudinal and multidimensional method. This provides an opportunity to apply some health behavior intervention techniques to promote patients to change their behavior and improve the effectiveness of chronic management results [50,55].

### Patient Behavior Preference Patterns

The behavior preference patterns of patients represent the individual inherent habits of patients in daily hypertension self-management [34-36], which is an essential factor to the design of management strategies. In this study, we found 3 behavior preference patterns of patients engaging in the pathway-based mHealth hypertension management, reflecting which patient groups preferred which type of behavior to achieve better self-management results. All of 3 patterns preferred PT-T, 1 preferred KL-T, and the other preferred SA-T, but there was no obvious preference for RO-T.

Patients with these 3 behavior preference patterns had significantly different demographic (gender and education level) and physiological (BP) characteristics. Compared with other patterns, patients with the preference for PT-T and KL-T had a lower education level, higher BP, and were much less likely to be male. First of all, there were more male patients than female patients, possibly because males were more inclined to use mHealth services for hypertension management [56,57]. However, we found that female patients were more willing to read knowledge content than male patients. Our finding is consistent with the finding by Al-Ansari et al [58] that oral health knowledge and health behavior were statistically significantly higher among the females than the males. Moreover, in this study, we found that patients with lower education level and poorly controlled BP were more likely to be highly involved in hypertension health education. Some scientific literature demonstrated a strong association between lower levels of education and poor health outcomes [59,60]. Health literacy was a potential pathway between levels of education and health outcomes [61]. Lee et al [62] and Nutbeam et al [63] also found that in comparison with people with higher education, people with lower education level were found to demonstrate lower health literacy and poor health. Naturally, these patients had a strong enthusiasm for learning about hypertension to promote their health literacy and improve health outcomes. The findings of patient preferences and characteristics are useful for designing personalized functions in the mHealth app to improve patient ability and engagement in hypertension self-management [34-36].

### Strengths and Limitations

The strengths of this study were that, first of all, the study used time series data to unveil the longitudinal and multidimensional behavioral characteristics of patient engaging in mHealth hypertension management, which contained the type, quantity, time spent, sequence, and transition probability of patient behavior. The findings help provide more precise timing of mHealth behavior interventions for hypertension management. To the best of our knowledge, there has been no literature that uses longitudinal data to describe the trails of patient multibehavior engagement in hypertension self-management. Secondly, we analyzed the demographic and physiological characteristics of patients with different behavior preferences. Our findings revealed the association between behavior preferences, demographics, and physiology. This provided information for the further design of the appropriate types of interventions for specific patient groups. Finally, we used data from real-world patients, which revealed patient behavior in the natural setting rather than attracting patients more likely to bear the burden of research-related evaluation. This helped to generate practical insights for the behavior of actual patients in daily hypertension management.

This study has limitations. One limitation was that the data were only from an mHealth hypertension app, which implies that the sample is presumably not representative of all patient groups. We also don’t know the behavior of patients who engage in self-management but not use the app, such as older patients who cannot use the mobile devices. Moreover, the behavioral characteristics of patients may be influenced and limited by the design of the app. Second, we used a first-order Markov chain, which simplified our analysis. Some other machine learning methods such as higher-order Markov chains should be applied to better represent global patient behavior. Finally, the behavior preferences can be caused by various demographic and social psychological characteristics of patients (such as marital status, profession, anxiety, depression, etc), so these factors need to be considered in the future studies.

### Conclusion

The mHealth services are an effective way to conduct out-of-hospital health care services for chronic disease. In this study, we have found 3 types of behavior transitions, 4 trails of patient multibehavior engagement, and 3 patient behavior preference patterns in the pathway-based mHealth hypertension self-management. These findings gained insights of actual behavior of patients in daily hypertension self-management. The behavioral characteristics of patients were dynamic, longitudinal, and multidimension, which may create opportunities to design tailored, personalized interventions within a specific behavior time to change patient’s behavior, develop positive behavioral habits, and continuously improve and maintain the effect of hypertension management.

## Appendix

Multimedia Appendix 1 Detailed information about behavioral states.

## Abbreviations

ANOVA: analysis of variance
BP: blood pressure
DBP: diastolic blood pressure
HR: heart rate
KL-T: knowledge learning trail
mHealth: mobile health
PT-T: perform task trail
RO-T: result-oriented trail
SA-T: support acquisition trail
SBP: systolic blood pressure
TP value: transition probability value
