# Supplementary material for: Exploring and Characterizing Patient Multibehavior Engagement Trails and Patient Behavior Preference Patterns in Pathway-Based mHealth Hypertension Self-Management: Analysis of Use Data
Source: JMIR Mhealth Uhealth. 2022 Feb 3;10(2):e33189. doi: 10.2196/33189 (PMC8855283; doi:10.2196/33189)
Supplement: Multimedia Appendix 1 [file mhealth_v10i2e33189_app1.docx]

**Detailed information about behavioral states**

| **Behavioral states** | **Description** |
| --- | --- |
| Main | The entrance to other behavioral states. |
| **Management plans** |  |
| Task BP | Measure blood pressure and record the blood pressure value and measurement time. |
| BP Guidance | Read how to measure blood pressure correctly. |
| BP History | View historical blood pressure data and trend graphs. |
| Task Drug | Take the medicine and record the name and time of the medicine. |
| Drug History | View medication history. |
| Task Food | Eat and record the name of the food and the time of the meal. |
| Food History | View the weekly staple food intake |
| Task Weight | Weigh and record weight and measurement time |
| Weight History | View the BMI trend. |
| Task Uncomfortable | Record the type and time of onset of uncomfortable symptoms. |
| **Reminder service** |  |
| Reminder | Choose from 5 types of tasks to set the task that needs to be reminded. |
| Reminder Item | Set reminder time and reminder type |
| **Health report** |  |
| Health Report | Read today's score, blood pressure, weight, steps, and the completion of the management plan. |
| Monthly Report | Read this month’s scores, the completion of the self-management plan, changes in blood pressure and weight. |
| **Leaderboard** |  |
| Ranking | View the current score and ranking of the management group. |
| **Health education** |  |
| Knowledge Content | Read an article about hypertension. |
| Knowledge List | View a list of all hypertension knowledge. |
| Knowledge Collect | View your own collection of hypertension knowledge. |
| Knowledge List by Tag | Choose the type of health education knowledge. |
| **Appointment** |  |
| Appointment | Choose the healthcare provider and hospital to make an appointment for consultation. |
| Show Appointment | Read the details of health managers and hospitals. |
| **Survey** |  |
| Questionnaire | Fill out the health questionnaire. |
| **Person information** |  |
| Person Information | View personal basic information. |
| Setting | Set daily push switch and push time, and pedometer switch. |
| Account | Set account name, ID number and mobile phone number. |
| **Login** |  |
| Login | Enter username and password to log in. |
| Register | Register a new account. |
